# MANTIS: an R package that simulates multilocus models of pathogen evolution

**DOI:** 10.1186/s12859-015-0598-9

**Published:** 2015-05-28

**Authors:** José Lourenço, Paul S Wikramaratna, Sunetra Gupta

**Affiliations:** Department of Zoology, University of Oxford, South Parks Road, Oxford, UK

**Keywords:** Multilocus, Pathogen, Evolution, Model, R package

## Abstract

**Background:**

In host-pathogen systems the development of immunity by the host places pressure on pathogens, by setting up competition between genetic variants due to the establishment of cross-protective responses. These pressures can lead to pathogen-specific, ubiquitous dynamic behaviours. Understanding the evolutionary forces that shape these patterns is one of the key goals of computationally simulated epidemiological models. Despite the contribution of such research methods in recent years to our current understanding of pathogen evolution, the availability of free software tools for the general public remains scarce.

**Results:**

We developed the **M**ultilocus **ANTI**genic **S**imulator (MANTIS) software package for the R statistical environment. MANTIS can simulate and analyse epidemiological time-series generated under the biological assumptions of the strain theory of host-pathogen systems by Gupta et al.

**Conclusions:**

MANTIS wraps a C/C++ ordinary-differential equations system and Runge-Kutta solver into a set of user-friendly R functions. These include routines to numerically simulate the system and others to analyse, visualize and export results. For this, the package offers its own set of time-series plotting and exportation functions. MANTIS’s main goal is to serve as a free, ready-to-use academic software tool. Its open source nature further provides an opportunity for users with advanced programming skills to expand its capabilities. Here, we describe the background theory, implementation, basic functionality and usage of this package. MANTIS is freely available from http://www.eeid.ox.ac.uk/mantisunder the GPL license.

**Electronic supplementary material:**

The online version of this article (doi:10.1186/s12859-015-0598-9) contains supplementary material, which is available to authorized users.

## Background

Epidemics of an infectious agent can be triggered by its introduction into a naive host-population, or by the evolution of novel antigenic types (strains) that evade herd-immunity created by circulating predecessors. The influenza A virus provides a much studied example: its surface proteins (antigens) are under strong selection by host immune responses and their molecular evolutionary shifts trigger recurrent epidemics and occasional pandemics [1,2]. In this context, a strain may be defined simply by the genetic loci that encode the antigens against which immune responses act, rather than by its entire genome. These potentially highly polymorphic sites may also be ranked by the degree to which the associated immune response influences the reproductive success of other strains [3,4].

The effects of immunity on pathogen evolution are best understood in the context of the ecology of host-pathogen systems: the development of immune responses to a pathogen alters the availability of the host as a resource for other pathogens which are targeted by similar responses. The outcome of this process of immune selection depends on the relative efficacy of immune responses against conserved and variable antigens, which in combination may prevent transmission by immune interference, resulting in the survival of a limited number of strains that may vary in time and space. For instance, many common childhood infectious diseases, such measles, mumps and whooping cough (pertussis), elicit strong immunity to conserved antigens, often leading to lifelong protection from future challenges. By contrast, protective immunity to conserved targets is either ineffective or is slow to develop against a number of other human pathogens such as the *Plasmodium falciparum* parasite (a causative agent of malaria) [5,6], the human immunodeficiency virus (HIV) [7] and several bacterial species such as *Neisseria meningitidis* and *Streptococcus pneumonaie*. This fundamental difference between pathogen types is evident in the area of vaccine development: we have mostly been successful in finding solutions against infections in which natural immunity to conserved targets is strong [8-10].

One way in which the evolution and dynamic behaviour of pathogens can be explored is through a multilocus mathematical framework, where each considered locus corresponds to an antigenic epitope, at which a fixed number of immunologically distinct variants can be expressed [11]. In practice, host-individuals who have been exposed to a particular antigen combination (strain) are assumed to acquire lifelong complete immunity to it, but also partial immunity to those strains expressing any shared antigens. By sheer existence of natural diversity, such antigen-sharing relationships can create a complex network of selective pressures, which may limit the emergence and circulation of some antigenic types and eventually structure the pathogen population into often observed patterns of diversity [11,12].

According to previous research based on this multilocus framework, the resulting pattern of diversity at equilibrium (strain structure) within a host-pathogen system is primarily determined by the strength of immune interference amongst antigenically related strains [11]. In the presence of low selection pressure, for instance, a lack of strain structure (NSS) is expected, in which antigenic variants can coexist at very similar prevalence levels. At intermediate levels of selection, however, cyclical/chaotic strain structure (CSS) emerges, where strains that dominate at a given point in time are constantly replaced by competing variants and measures of single-strain dominance (SSD) are found to be high. For even stronger immune selection, the cyclical behaviour is suppressed, giving rise to a discrete strain structure (DSS) in which strains self-organise into groups with non-overlapping (discordant) antigen repertoires that minimize competition and ease co-existence.

Other host-pathogen frameworks have alternatively shown that strain dynamics and associated structures can be represented by *continuous strain spaces* (e.g. one-dimensional arrays) in which strains are assumed to be antigenically related to a restricted subset of variants in the neighbouring antigenic space [13-15]. In these frameworks, similarly to the multilocus approach, competition for hosts can cause the pathogen population to become polarized within such continuous spaces in a manner analogous to *character displacement* across an ecological resource (e.g. specialisation on seed/beak size among coexisting species of birds). These models are therefore in agreement that strong immune selection favours the evolution of antigenically distinct types minimizing immunological interference that tend to present DSS-like population dynamics.

The existence of distinctly measurable strain structures in host-pathogen systems is of interest to epidemiologists as the implications for the control of infectious disease are considerable. For instance, the natural propensity to exhibit CSS-like dynamics, as is the case for influenza A viruses, can complicate and restrict our ability to interpret and predict the outcome of interventions such as vaccination [1,2,16]. At the same time, the observation of DSS implies that genomic regions under strong immune selection should be detectable, for instance through linkage disequilibrium, which is of paramount importance for research and discovery of potential targets for vaccines and antipathogen drugs [17-19].

Despite the contribution of Gupta et al. multilocus strain theory [1,12,17-21] and other *strain theories*[2,13-15] in recent years to our current understanding of the population dynamics and evolution of multi-strain pathogens, their biological simplicity is often overshadowed by inherent mathematical and computational complexities. Moreover, the availability of free software to explore the proposed framework alternatives has not grown at the same rate as this diversity in knowledge. This is a particularly important point in light of an increasing use of computation in biological and ecological studies, to which simple and openly available tools may contribute immensely. Here, we present and describe a new R package aiming to reduce the existing gap between mathematical approaches and population biology education and research.

## Implementation

The **M**ultilocus **ANTI**genic **S**imulator (MANTIS) package was designed for the R statistical environment by the Evolutionary Ecology of Infectious Disease (EEID) research group of the Department of Zoology at the University of Oxford. MANTIS is a tool to simulate and analyse epidemiological time-series, generated under the biological assumptions of the strain theory of host-pathogen systems by Gupta et al. [11].

### Environment

R is a language and environment for statistical computing and graphics, freely available for download and general use [22]. This platform was chosen for its open-source nature, general acceptance within the computational and bioinformatics communities, connection to educational programmes and ease for data manipulation and graphical rendering. The core of MANTIS is essentially a dynamic system of ordinary-differential equations (see below) and a fixed-step Runge-Kutta solver [23]. For computational performance reasons, these were implemented using C/C++ code. MANTIS therefore requires Rcpp - a free R package aiming at providing seamless integration of R and C/C++ [24]. All code was designed and implemented in Ubuntu 14.04 LTS (Trusty Tahr), using R version 3.0.2, as well as *g++* version 4.8.2 and Rcpp version 0.11.2. We refer the reader to the section Availability and Requirements at the end of this article for a summary of the software involved in this version of MANTIS.

### Multilocus framework

**Figure 1:**
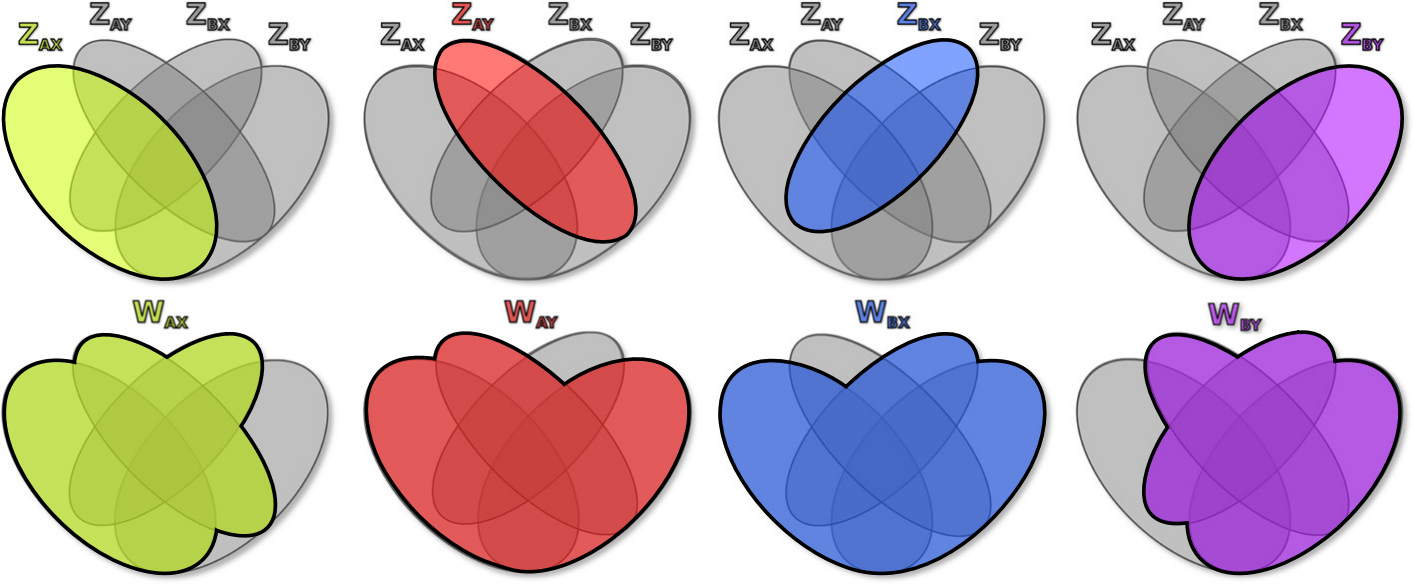
Schematic example of a host-pathogen system within the multilocus framework. A schematic of overlapping compartments is used to indicate the proportions immune to each strain (*Z*) and to antigenically related variants (*W*), from which the proportion of infectious (*Y*) can be deduced (for simplicity, a {2,2} antigenic system is presented, see main text for model equations). The background grey Venn diagrams present all the possible overlaps between the *Z* and *W* subclasses. Colours are used to highlight the sections of the diagrams related to each strain: *AX* in green, *AY* in red, *BX* in blue and *BY* in purple. On the top row, *Z* classes are presented. On the bottom row *W* classes are presented, which include the intersection of multiple *Z* classes according to the allele-sharing relationships between strains.

The system of ordinary-differential equations (ODEs) below summarizes the basic model structure implemented within the package MANTIS (for more general mathematical formulations see for example [11,12,25]). For simplicity, a host-pathogen system with two relevant loci and two possible alleles is here described - it is considered that alleles {*A*,*B*} can occur at the first locus and {*X*,*Y*} at the second. Similarly to what has been done in previous publications, a system of overlapping compartments can be used to represent the proportions of hosts belonging to each relevant, epidemiological class (Figure 1).

(1) $$\begin{array}{*{20}l} &\frac{{dZ}_{AX}}{dt} =\beta Y_{AX} (1-Z_{AX}) - \mu Z_{AX} \end{array} $$

(2) $$\begin{array}{*{20}l} &\frac{{dW}_{AX}}{dt} = \sum_{ij-AX}^{} \beta Y_{ij} (1-W_{AX}) - \mu W_{AX} \end{array} $$

(3) $$\begin{array}{*{20}l} & \frac{{dY}_{AX}}{dt} \,=\, \beta Y_{AX} ((1\!-W_{AX}) \,+\, (1\,-\,\gamma)(W_{AX}\,-\,Z_{AX})) -\sigma Y_{AX} \end{array} $$

According to this strain theory, it is assumed that host-individuals who have been exposed to a particular pathogen strain (*Z*_*AX*_, exemplifying for strain *AX*) are immune for life to further challenge by the same strain, while those who have been exposed to strains that share alleles with it (*W*_*AX*_) have reduced probability (*γ*) of transmitting the strain once infected (*Y*_*AX*_). The parameter *γ* therefore reflects the strength of antigen-specific responses in transmission prevention. The parameters *β* and 1/*σ*, respectively, define the transmission coefficient and infectious period of the pathogen, while 1/*μ* corresponds to the life expectancy of the host population. Here, the notation *i* *j*−*A* *X* indicates all strains sharing alleles with strain *AX*.

Using MANTIS, the antigenic structure here exemplified can be easily generalized to multiple loci with any level of diversity. We propose and will henceforward use the notation {3,4,10}, for example, to indicate a system with three loci {*i*,*k*,*j*} and *N*_*i*_=3, *N*_*k*_=4 and *N*_*j*_=10 alleles, respectively. Finally, it is important to note that for any given structure, the total number of possible strains is *N*_*i*_×*N*_*k*_×… *N*_*n*_.

### Key features and functionalities

MANTIS wraps a significant amount of C/C++ and R programming into a set of user-friendly R functions. These include the routine (*runMANTIS*) to simulate the complex systems described above but also others to analyse, visualize and export results. For this, the package offers its own set of plotting functions (*plotY, plotYDiversity, etc.*) that support users unfamiliar with the R environment from the need to explicitly program time-series extraction, transformation, formatting and displaying. The same is done for exportation of time-series, for which functions (*exportY, exportYDiversity, etc.*) are offered to write data into text-files containing comma-separated values (CSV), generally usable in spreadsheet-office applications. With the goal of facilitating similar analyses to what has already been published in research articles based on this strain theory, MANTIS also offers routines to obtain quantifications of interest on the simulated time-series. These range from calculations of diversity (*calcDiversity*) using standard ecological methods such as the Shannon Index [25], to epidemiological measures of single-strain dominance (*calcSingleStrainDominance*) [1]. The detailed description of each available method and associated routines within MANTIS escapes the scope of this article and new versions of the package are expected to become available in the near future. Hence, for updated references and detailed explanations that may include the mathematical formulations of built-in methods, we refer the reader to the package’s Manual, available for download at the EEID’s website [26].

## Results and discussion

**Figure 2:**
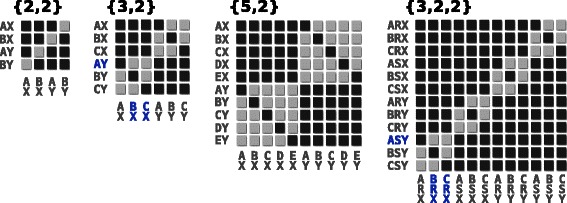
Examples of strain allele-sharing relationships within the multilocus framework. A set of matrix-diagrams highlight the relationships between all possible strains within systems defined with different antigenic structures. Relationships are highlighted in dark grey squares, representing pairs of strains (row, column) that share at least one allele (and therefore antigen). In contrast, light grey squares mark pairs with non-overlapping allele repertoires (discordant) that share no antigens. Examples of discordant strains are shown in blue for the systems {3,2} (*AY* with *BX* and *CX*) and {3,2,2} (*ASY* with *BRX* and *CRX*). Letters were arbitrarily chosen to build figurative strain sequences (locus 1: A, B, C, D, E; locus 2: R, S; and locus 3: X and Y).

The package MANTIS introduces no restrictions to the complexity of the system to be simulated. However, due to the steep increase in the number of ODEs required for more complex antigenic structures, computational limitations may be present for the user. Figure 2 presents some examples of the versatility of Gupta’s et al. framework, for systems with 4, 6, 10 and 12 possible strains. From this simple illustration it is possible to note how different antigenic structures will manifest as a multitude of relationships between strains and dissimilar networks of cross-immunity. For instance, the systems {3,2} and {3,2,2} have very different numbers of total strains (6 and 12), but each existing strain is only discordant with two others (exemplified in blue). Likewise, the systems {5,2} and {3,2,2} have similar number of strains (10 and 12) but the full level of discordance is verydifferent.

**Figure 3:**
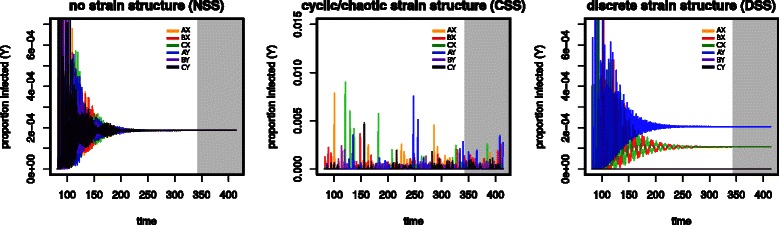
Examples of dynamic solutions with different strain structures at equilibrium. Dynamic solutions obtained from MANTIS, using three levels of cross-immunity *γ*=0.10*(left)*, *γ*=0.75*(centre)* and *γ*=0.95*(right)*, within an antigenic system with structure {3,2} and 6 strains. The level of immune selection is here seen to structure the pathogen population into NSS, CSS, and DSS. Examples are initialized with the same initial conditions and parameters are *β*=292, *σ*=(1/5)∗365 and *μ*=1/50, resulting in a basic reproduction number (*R*0) of ≈4. Grey shaded areas highlight the dynamics at equilibrium. The beginning of the dynamic series is discarded for visualization purposes. Letters were arbitrarily chosen to build figurative strain sequences (locus 1: A, B; locus 2: X and Y). The minimal R code required to replicate these dynamics can be found in the Additional file 1.

To exemplify the simulation potential of MANTIS, we further explore the population dynamics of example {3,2}. We choose this system for simplicity and visualization purposes, given its small strain size (2 loci) and number (6 strains). While we focus this example on the dynamics of the infected portion (*Y*) of the host population, it should be noted that with MANTIS it would also be possible to analyse the cross-protected (*W*) and immune (*Z*) portions. In Figure 3 it is shown how the strength of immune selection (*γ*) dictates the transient dynamics and strain structure that is reached at equilibrium. When cross-immunity between strains sharing alleles is low (Figure 3, left), NSS is observed as strains co-exist and share similar prevalence levels. In contrast, as immune pressure is increased, the dynamic state at equilibrium changes and strain structures emerge. For example, at a reasonably high *γ*=0.75, CSS is presented (Figure 3, centre), in which strains frequently replace each other in time through incomplete exclusion arising from temporary competition for susceptible hosts. This dynamic behaviour provides a basis for understanding the population dynamics of pathogens such as influenza A [1,12], as well as the within-host dynamics of HIV and *Plasmodium falciparum* [21], which are all characterised by the sequential appearance of novel antigenic types in time. At even higher levels of selection, however, the cyclical behaviour is suppressed and DSS emerges, in which subsets of strains appear present at different prevalence levels (Figure 3, right). In this case, given that strains have no explicit epidemiological asymmetries in the framework, initial conditions dictate which subset becomes dominant. Nevertheless, it is the strong immune selection that forces variants to segregate, given their allele-sharing profiles. In this example, strain *AY* dominates the population, followed by *BX* and *CX*, while strains *AX*, *BY* and *CY* are virtually extinct. It is straightforward to note that under this regime of strong immune pressure, the persistent subsets are discordant (*AY* shares no alleles with { *B* *X*,*C* *X*}), which arises from the natural aptitude of the strains to organize into subsets that minimize competition. Concurrently, all strains that are driven to extinction necessarily share alleles with the highly prevalent ones. The best known examples of this form of strain structure have been found among the hypervariable regions of subcapsular antigens of the bacterial species *Neisseria meningitidis*: non-overlapping associations between the VR1 and VR2 regions and variants of the iron-regulated outer membrane protein FetA are evident in the global isolate collection represented in the PubMLST database [27]. Similar patterns have also been observed among the Opacity-associated outer membrane proteins of the *meningococcus*, which play a role in adhesion to the human host tissue during colonisation and invasion [17,28].

**Figure 4:**
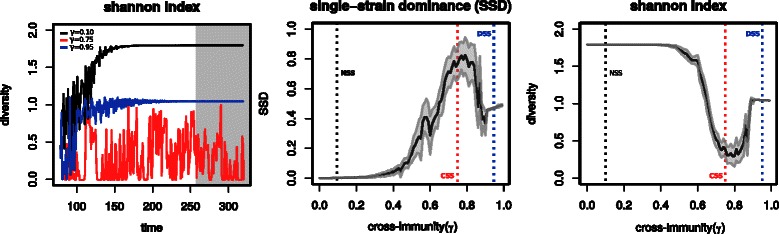
Examples of strain diversity solutions for different strain structures. Dynamic solutions generated by MANTIS for an antigenic system with structure {3,2} and 6 strains. *(left)* Strain diversity (Shannon Index), for three levels of cross-immunity *γ*=0.10 (black), *γ*=0.75 (red) and *γ*=0.95 (blue). Examples correspond to series presented in Figure 3 for the same *γ* values. Grey shaded area highlights the dynamics at equilibrium used for the remaining plots. *(centre, right)* Single-strain dominance (SSD) and strain diversity (Shannon Index) for different values of *γ*. Grey lines and areas are the mean and standard deviation, respectively. Variation is obtained by running 100 simulations with random initial conditions for each *γ* value. Vertical lines mark the *γ* regimes used in Figure 3 and this figure, left. The minimal R code required to replicate these dynamics can be found in the Additional file 1.

The manner in which strains are found to be organized in host-pathogen systems further dictates the patterns and levels of strain diversity that can be found in time [1,12]. To complete the examples presented in Figure 3 we further demonstrate how diversity measures can be obtained from the inbuilt functionality of MANTIS (Figure 4). As expected, when selection is virtually absent (*γ*=0.1) and strains self-organize to co-circulate at similar prevalence levels (NSS), diversity is maximized. In contrast, in regimes in which cross-immunity plays a role (CSS and DSS) and strains suffer competition, diversity is often lower (Figure 4, right). In the case of DSS this arises from the competitive exclusion of some strains, while in CSS it is a consequence of recurrent replacement and temporary dominance of subsets of strains (Figure 3, centre, right). Importantly, the actual patterns of diversity in time are dependent on the inherent dynamic behaviour of the strains. This can be seen in the left subplot of Figure 4, in which the regime defined by *γ*=0.75 and characterized by CSS (Figure 3, centre) also presents oscillating diversity patterns. Another consequence of such fluctuating behaviour in strain prevalence can be seen in measures of single-strain dominance, which tend to be maximized in the presence of CSS regimes (Figure 4, centre).

## Conclusions

The development of immunity amongst hosts places pressure on pathogens by setting up competition between genetic variants, since a previously infected host may no longer be available for infection due to the establishment of responses that are (partially) effective against a variety of other strains. This sort of competition is particularly acute when the principal targets of immunity are conserved antigens shared by all strains: in these cases, each host can only sustain a single infection by a particular pathogen species, as is the case with many common childhood infections such as measles, mumps and pertussis. In contrast, when the principal targets of immunity are variable, the same principles of competitive exclusion can lead to cyclical/chaotic fluctuations in the frequencies of different strains, or in the coexistence of strains which differ as widely as possible in the immune responses they provoke in order to relax competition and associated selective pressures. The observation and study of distinct strain structures in a specific host-pathogen system are of paramount importance to epidemiologists as the implications for control areconsiderable.

Understanding the evolutionary forces that shape the observed structures of pathogen populations is one of the key goals of computationally simulated epidemiological models. The strain theory herein described states that a pathogen’s infection history and its future potential are shaped by natural selection arising from host immune pressures [11]. The application of such concept was herein exemplified in a basic study case. This consisted in the definition of a relevant antigenic structure and simulation of population dynamics and expected levels of strain diversity. The main goal of this example was to highlight the fundamental functionalities of the MANTIS package, with no specific pathogen in mind. Nonetheless, this same mathematical approach has been previously used to research specific viral and bacterial agents, such as the malaria parasite *Plasmodium falciparum* [21], the influenza A virus [1,12,20] and *Neisseria meningitidis* [17,18,28].

MANTIS’s main function is to serve as a free academic software tool. We argue for this software’s place and usefulness in educational programmes such as epidemiology and ecology courses, by supporting students in their exploration of host-pathogen systems in which host immunity may play a significant role. With this in mind, the package was developed to be user-friendly, not only wrapping the use of complex ODE systems into single-function calls, but also including a variety of functions for easy time-series visualization and exportation. The open source nature of MANTIS further provides a valuable opportunity for researchers with advanced programming skills to expand and tweak its capabilities for their own research goals. In fact, as the number of research articles based on this strain theory grows, we predict that many new functionalities will have a place on the base content of this package. A future intended direction is therefore to update and republish this software regularly on the Evolutionary Ecology of Infectious Disease group’s website [26].

## Availability and requirements

MANTIS has a dedicated manual that follows the well-established format for R packages. It presents a general description of the package’s functionality, as well as examples, a short introduction and key literature references for the strain theory herein described. As new versions of the package are expected in the near future, the reader should refer to this manual for updated references and mathematical formulations of MANTIS’s inbuilt methods. The package can be installed using a platform-independent, source-based file (note: compiling source packages may require the pre-installation of Rtools [29] on Windows machines). Both the manual and source file can be found at the EEID’s website for download [26]. Newer versions and related materials will be deposited on this website and we refer the reader to it for further information and future changes. Finally, the following list summarizes key information about the version of MANTIS explored and described in this article:

- **Project name:** Multilocus ANTIgenic Simulator (MANTIS).
- **Creators:** EEID group at University of Oxford.
- **Current Version:** 1.0 (cryptonym: *green egg*).
- **Project home page:** http://www.eeid.ox.ac.uk/mantis
- **Manual, examples and help:** Available at the project’s website.
- **Operating system:** Platform independent.
- **License:** GNU GPL.
- **Programming languages:** R, C, C++.
- **Other requirements:** R 3.0 or higher, Rcpp 0.11.2 or higher, GNU GCC 4.8.2 or higher, Rtools.

## Additional file

Additional file 1
**Minimal code for examples in the main text.** The minimal R code necessary to replicate the dynamics of Figures 3 and 4, using MANTIS, can be found in this PDF file.
