# Supplementary material for: MANTIS: an R package that simulates multilocus models of pathogen evolution
Source: BMC Bioinformatics. 2015 May 28;16:176. doi: 10.1186/s12859-015-0598-9 (PMC4445977; doi:10.1186/s12859-015-0598-9)
Supplement: Additional file 1 — Minimal code for examples in the main text. The minimal R code necessary to replicate the dynamics of Figures 3 and 4, using MANTIS, can be found in this PDF file. [file 12859_2015_598_MOESM1_ESM.pdf]

Minimal code for examples in the main text.

Here we demonstrate the package's usage by presenting a set of minimal R instructions that can reproduce the population dynamics presented in figures 3 and 4. All used functions are either included in MANTIS or present by default in a basic installation of R.

```
#load package MANTIS
library(MANTIS)

###################################################
### minimal code for figure 3

#antigenic structure: with 2 loci (6 strains)
epiStruc <- c(3,2)

#get number of strains in the system
nStrains <- extractNumberStrains(epiStruc)

#time at which the solver will stop
tMax <- 415

#solver step
tInt <- 0.005

#record solution every tObsPer steps
tObsPer <- 150

#random initial conditions for infected (Y)
infInitCond <- c(4.307817e-05, 6.552645e-05, 6.619973e-05,
                1.698892e-05, 2.037509e-05, 2.093472e-05)

#parameter values here defined by year (output time therefore in years)
beta <-  292
sigma <-  (1/5)*365 #5 days infectious period
mu <-  1/50 #50 years of life-span

#colours for the 6 strains
cols <- rev(c("black","purple","blue","limegreen","red","orange"))

#running model with weak cross-immunity
gamma <- 0.1
simdata <- runMANTIS(epiStruc, tMax, tObsPer, tInt, infInitCond, beta, gamma, sigma, mu)

#plot infected class (left, figure 3)
plotY(simdata, xiObs=0.2, xfObs=1, ymax=0.0007, colours=cols, yylab="Y", mainRight="NSS")

#running model with intermediate cross-immunity
gamma <- 0.75
simdata <- runMANTIS(epiStruc, tMax, tObsPer, tInt, infInitCond, beta, gamma, sigma, mu)

#plot infected class (centre, figure 3)
plotY(simdata, xiObs=0.2, ymax=0.015, colours=cols, yylab="Y", mainRight="CSS")

#running model with strong cross-immunity
gamma <- 0.95
simdata <- runMANTIS(epiStruc, tMax, tObsPer, tInt, infInitCond, beta, gamma, sigma, mu)

#plot infected class (right, figure 3)
plotY(simdata, xiObs=0.2, xfObs=1, ymax=0.0007, colours=cols, yylab="Y", mainRight="DSS")

###################################################
### minimal code for figure 4

#define epitope structure, with 2 loci (6 strains)
epiStruc<- c(3,2)
```

```
#get number of strains in the system
nStrains<- extractNumberStrains(epiStruc)

#time at which the solver will stop
tMax<- 415

#solver step
tInt<- 0.005

#record solution every tObsPer steps
tObsPer<- 150

#random initial conditions for infected
infInitCond <- c(4.307817e-05, 6.552645e-05, 6.619973e-05,
                1.698892e-05, 2.037509e-05, 2.093472e-05)

#parameter values here defined by year
#thus output time scale will be years
beta<-  292
sigma<-  (1/5)*365 #5 days infectious period
mu<-  1/50 #50 years of life-span

#running model with weak cross-immunity
gamma<- 0.1
simdata<- runMANTIS(epiStruc, tMax, tObsPer, tInt, infInitCond, beta, gamma, sigma, mu)

#plot infected class (left, black, figure 4)
plotYDiversity(simdata, xiObs=0.2, xfObs=1, ymax=2, colour="black", yylab="Y",
mainRight="shannon index, diversity in NSS")

#running model with intermediate cross-immunity
gamma<- 0.75
simdata<- runMANTIS(epiStruc, tMax, tObsPer, tInt, infInitCond, beta, gamma, sigma, mu)

#plot infected class (left, red, figure 4)
plotYDiversity(simdata, xiObs=0.2, xfObs=1, ymax=2, colour="red", yylab="Y", mainRight="shannon
index, diversity in CSS")

#running model with strong cross-immunity
gamma<- 0.95
simdata<- runMANTIS(epiStruc, tMax, tObsPer, tInt, infInitCond, beta, gamma, sigma, mu)

#plot infected class (left, blue, figure 4)
plotYDiversity(simdata, xiObs=0.2, xfObs=1, ymax=2, colour="blue4", yylab="Y",
mainRight="shannon index, diversity in DSS")

#set a wide range of gammas to calculate single-strain dominance
gammas<- c(0.1, 0.2, 0.3, 0.4, 0.5, 0.6, 0.75, 0.8, 0.95, 0.99)

#run model for each gamma value and get SSD for each run
y.SSDs<- measureSSDfromGammaRange(gammas, method="host", epiStruc, tMax, tObsPer, tInt, tStep,
infInitCond, beta, sigma, mu, xiObs = 0.7, xfObs = 1)

#plot gammas against SSDs found (centre, figure 4)
plot(gammas, y.SSDs, t='l', col="grey44")

#set a wide range of gammas to calculate strain diversity
gammas<- c(0.1, 0.2, 0.3, 0.4, 0.5, 0.6, 0.75, 0.8, 0.95, 0.99)

#run model for each gamma value and get shannon index for each run
y.DIVs<- measureDiversityfromGammaRange(gammas, method="shannon", epiStruc, tMax, tObsPer, tInt,
tStep, infInitCond, beta, sigma, mu, xiObs = 0.7, xfObs = 1)

#plot gammas against DIVs found (right, figure 4)
plot(gammas, y.DIVs, t='l', col="grey44")
```
